# IGZO-Based Electronic Device Application: Advancements in Gas Sensor, Logic Circuit, Biosensor, Neuromorphic Device, and Photodetector Technologies

**DOI:** 10.3390/mi16020118

**Published:** 2025-01-21

**Authors:** Youngmin Han, Juhyung Seo, Dong Hyun Lee, Hocheon Yoo

**Affiliations:** 1 Department of Semiconductor Engineering, Gachon University, Seongnam 13120, Republic of Korea; ym708@gachon.ac.kr; 2 Department of Electronic Engineering, Gachon University, Seongnam 13120, Republic of Korea

**Keywords:** IGZO, gas sensor, logic circuit, bio sensor, neuromorphic device, photodetector

## Abstract

Metal oxide semiconductors, such as indium gallium zinc oxide (IGZO), have attracted significant attention from researchers in the fields of liquid crystal displays (LCDs) and organic light-emitting diodes (OLEDs) for decades. This interest is driven by their high electron mobility of over ~10 cm^2^/V·s and excellent transmittance of more than ~80%. Amorphous IGZO (a-IGZO) offers additional advantages, including compatibility with various processes and flexibility making it suitable for applications in flexible and wearable devices. Furthermore, IGZO-based thin-film transistors (TFTs) exhibit high uniformity and high-speed switching behavior, resulting in low power consumption due to their low leakage current. These advantages position IGZO not only as a key material in display technologies but also as a candidate for various next-generation electronic devices. This review paper provides a comprehensive overview of IGZO-based electronics, including applications in gas sensors, biosensors, and photosensors. Additionally, it emphasizes the potential of IGZO for implementing logic gates. Finally, the paper discusses IGZO-based neuromorphic devices and their promise in overcoming the limitations of the conventional von Neumann computing architecture.

## 1. Introduction

Since IGZO was first developed in 2004 by Prof. Hideo and his team at Tokyo Institute of Technology, it has garnered significant attention and growth [1]. His team focused on IGZO’s composition of indium (In), gallium (Ga), zinc (Zn), and oxygen (O), which offered high electron mobility [2,3] and transparency—crucial breakthroughs for high-performance displays, especially in LCDs and OLEDs [4,5,6]. IGZO achieved great commercial success, with Sharp becoming the first to produce LCD panels using IGZO-TFT technology in 2012, improving the aperture ratio by up to 20% and reducing power consumption due to IGZO’s high mobility and low off-current [7,8,9]. IGZO-TFT has since been used in smartphones, tablets, high-resolution notebooks, gaming laptops, and OLED TVs. Compared to zinc oxide, IGZO’s advantage lies in its ability to be deposited in a uniform amorphous phase while maintaining high carrier mobility with minimal photosensitivity in the ultraviolet range, making fully transparent transistors possible.

IGZO can be synthesized using a range of thin-film deposition techniques such as sputtering, atomic layer deposition (ALD) [10,11], pulsed laser deposition (PLD) [12,13], and chemical vapor deposition (CVD) [14,15]. By precisely controlling the ratios of In, Ga, Zn, and O during deposition, it is possible to tailor the channel properties to meet the requirements of specific applications. Typically, the electron mobility is influenced by the ratios of In and Zn, while the device stability is determined by the proportions of Ga and O [16]. These methods enable precise control over film characteristics, making IGZO particularly suitable for large-area deposition and the fabrication of large-scale electronic devices [17]. Its exceptional electronic properties—such as high electron mobility (>10 cm^2^∙V·s) and ultra-low off-current levels (down to the femtoampere range)—and compatibility with bottom-up fabrication processes have garnered significant interest. Initially developed for advanced display technologies, such as OLEDs and thin-film transistor (TFT)-LCDs, IGZO is now being explored for next-generation applications, including neuromorphic computing, transparent electronics, and flexible devices.

IGZO stands out as a versatile material due to its compatibility with large-area processing and its ability to be deposited onto various substrates, regardless of their form factor, through bottom-up fabrication techniques. Bottom-up fabrication techniques enable the deposition of gate electrodes, insulating layers, channel materials, and source/drain electrodes sequentially onto a substrate, allowing device fabrication on a variety of substrates. This approach eliminates the need for additional processes, such as etching, by integrating patterning and deposition in a single step, thereby enhancing compatibility with diverse substrate types [18,19,20]. This versatility allows IGZO to serve as an active layer in a wide range of applications. Moreover, IGZO’s functionality extends to phototransistors, enabling novel applications such as optically induced neuromorphic computing devices [21,22] and in-sensor computing systems [23,24] utilizing light pulses.

Another merit of IGZO is that its high process compatibility supports its use on diverse substrates, including glass, silicon, and polymers. Its low-temperature processing capability enhances its suitability for gas sensor materials [25,26], offering high selectivity. The material’s superior chemical resistance prevents device degradation from repeated exposure to gas molecules, contributing to extended device lifespans. Additionally, its exceptional thermal stability ensures reliable operation in high-temperature environments without damage to its semiconductor properties.

The high electron mobility of IGZO plays a critical role in biosensing applications, where it enables rapid and precise detection of signals generated by interactions with biomolecules (e.g., proteins, DNA, and antigen–antibody complexes) [27,28]. This high mobility allows IGZO-based sensors to detect extremely low concentrations of biomolecules, resulting in an exceptionally low limit of detection. Furthermore, the fast signal processing capability of IGZO is highly advantageous for real-time diagnostics. Its low power consumption makes it an ideal candidate for portable biosensors and battery-operated devices. This feature is particularly valuable for medical devices requiring long-term monitoring, ensuring sustained operation without frequent battery replacement. In this context, this paper explores cutting-edge advancements in IGZO-based applications, aiming to expand their scope and impact.

**Figure 1:**
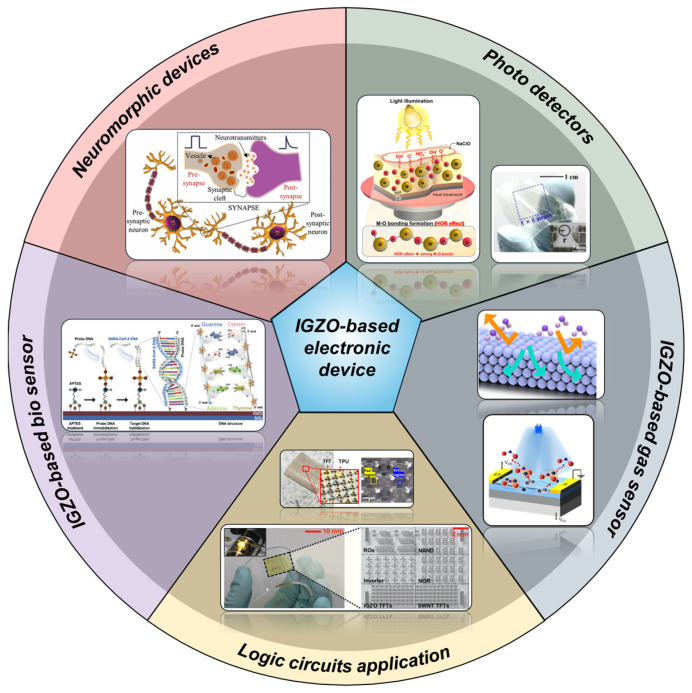
Schematic of IGZO-based electronic device applications. Reproduced with permission [29,30,31,32,33,34,35,36]. Copyright 2020, American Chemical Society; Copyright 2021, Elsevier B.V.; Copyright 2021, American Chemical Society; Copyright 2021, Wiley-VCH GmbH. (Reprint by permission for John Wiley & Sons); Copyright 2024, Elsevier; Copyright 2020, Wiley-VCH GmbH. (Reprint by permission of John Wiley & Sons); Elsevier; Copyright 2020; Copyright 2021, American Chemical Society.

From this perspective, this paper presents a comprehensive overview of the applications of IGZO, a metal-oxide semiconductor, as shown in Figure 1. Based on the versatile capabilities of IGZO, Part 1 summarizes the gas sensors that can detect various gases. In Part 2 focuses on the results of multi-value logic (MVL) based on IGZO and the implementation of complex logic circuits. Part 3 summarizes the process of detecting biomaterials by antigen–antibody reactions. A case study on the application of synaptic devices utilizing IGZO is provided in Part 4. Part 5 presents the implementation results of IGZO-based photo detectors.

## 2. IGZO Semiconductor-Based Electronic Applications

### 2.1. IGZO-Based Gas Sensors

Global air pollution has been accelerated by promotion from automobiles and industrial activities resulting from rapid industrialization and urbanization. Specifically, various gases such as nitrogen oxides (NO_x_) [37,38,39], ozone (O_3_) [40,41,42], and ammonia (NH_x_) [43,44,45] can negatively impact human health and air quality. Therefore, the demand for gas sensors that enable real-time monitoring of air pollution and gas leaks from factories and effective response strategies has increased. In the case of gas sensors, it is essential that gas molecules are absorbed and desorbed effectively. To detect the conductive differences arising from the adsorption and desorption processes of gas molecules [46,47], gas sensors can be made to operate with high sensitivity. Therefore, it is essential to have semiconductors that can operate stably at room temperature, are cost-effective, and can reliably perform stable and repeatable gas adsorption and desorption processes [48]. Various oxide semiconductors have garnered interest from researchers due to their efficiency in air stability and gas detection [49,50,51,52,53]. Notably, IGZO is recognized for its stable performance at room temperature and its ability to maintain strong and reliable characteristics even through adsorption and desorption processes. As a result, numerous studies have reported on gas sensors utilizing IGZO semiconductors [30,54,55,56].

**Figure 2:**
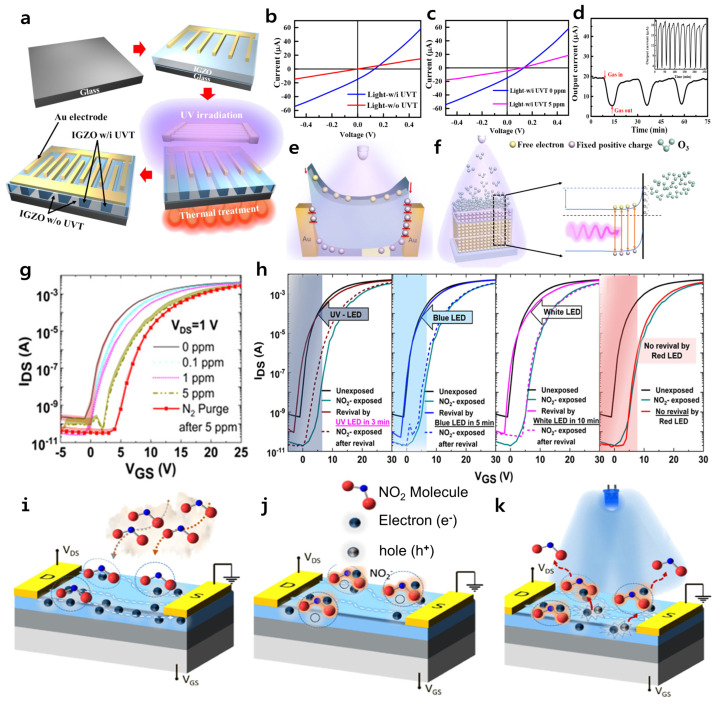
(**a**) Schematic illustration of the manufacturing procedure for self-powered O₃ gas sensor fabrication. (**b**) I–V characteristics under UV illumination (25 mW/cm^2^) at 0 V bias for gas sensors with and without UVT. (**c**) Illuminated I–V characteristics for a gas sensor with UVT in the presence and absence of 5 ppm O₃ gas. (**d**) The gas response characteristics under continuous UV illumination (25 mW/cm^2^) at 0 V during repeated exposure to 5 ppm O₃ gas. (**e**) Energy band diagrams of a sensor exhibiting an asymmetrical Schottky barrier height at 0 V, shown under UV illumination. (**f**) Schematic diagram depicting the adsorption process under UV illumination, along with the energy band interaction with O₃ molecules at the surface. Reproduced with permission. Ref. [57] Copyright 2022, American Chemical Society. (**g**) Gas response characteristic of IGZO TFT-based sensors after 3 min of exposure to NO_2_ concentrations ranging from 100 ppb to 5 ppm. (**h**) The gas response characteristics of IGZO TFT-based gas sensors exposed to NO_2_ show recovery under illumination from various commercial LEDs of different wavelengths, using the UV LED (400 nm) for 3 min, the blue LED (450 nm) for 5 min, and the white LED for 10 min, but no recovery occurs after 15 min with the red LED (635 nm). A schematic diagram of the sensing and revival mechanisms: (**i**) adsorption of NO_2_ molecules, (**j**) depletion of surface charge carriers, and (**k**) the device revival mechanism under LED illumination. Reproduced with permission. Ref. [29] Copyright 2020, American Chemical Society.

In 2022, Huang et al. implemented a self-powered O_3_ gas sensor with a metal–semiconductor–metal (MSM) structure (Figure 2a) [57]. This device is designed to operate in a self-powered operation by forming an asymmetric Schottky barrier through ultraviolet treatment (UVT). This induces the photovoltaic effect, enabling self-powered operation. Applying light energy to a gas sensor can provide more electrons in the conduction band to promote adsorption and desorption processes, and also increase its sensitivity to gas detection [58,59,60]. Therefore, the fabricated devices were investigated for their I–V characteristics under ultraviolet (UV) light. For the device without UVT, due to the symmetrical Schottky barrier, the device exhibited symmetrical current characteristics, and the photovoltaic effect could not be observed (Figure 2b). However, the device with UVT showed an asymmetric I–V curve, indicating the occurrence of the photovoltaic effect, confirming that UVT is essential for self-powered operation. Furthermore, the I–V characteristics were investigated when 0 ppm and 5 ppm of O_3_ gas were applied to the UVT-exposed devices (Figure 2c). When subjected to an O_3_ gas concentration of 5 ppm, the device with O_3_ molecules adsorbed on IGZO showed reduced conductivity compared to the unexposed device. However, when gas inflow stopped, O_3_ molecule desorption occurred from the IGZO surface, resulting in increased conductivity (Figure 2d). The reason for the decrease in conductivity upon gas inflow is that O_3_ molecules are adsorbed on the IGZO surface, electrons are trapped by the adsorbed O_3_ molecules, and the concentration of electrons utilized as carriers decreases [61,62,63].

The detailed gas detection mechanism proposed by the authors was as follows. Under UV light exposure, the electron–hole pairs become separated by the internal electric field in the Au/IGZO junction. In the case of hole carriers, the hole-trapping density at the negative electrode without UVT is higher than at the positive electrode, leading to holes being mainly trapped at the defects in the negative electrode, a phenomenon known as the hole-trapping effect. Therefore, the trapped hole carriers cause the height of the Schottky barrier at the negative electrode to be lower than that at the positive electrode (Figure 2e). Subsequently, when exposure O_3_ gas is released and adsorbed on the surface of IGZO, the electron concentration decreases as electrons bind to the surface adsorbed O_3_ molecules, and this region forms a depletion layer that lacks charge carriers. In the depletion layer, the concentration of free electrons decreases, which reduces the electron concentration within the semiconductor, causing the Fermi level to shift lower. As a result, the band structure of the semiconductor is bent up and reorganized to achieve electrical equilibrium, resulting in a decrease in conductivity.

Vijjapu et al. reported a bottom gate and top contact structure-based IGZO TFT-type NO_2_ detection sensor [29]. The electrical characteristics of the IGZO TFT-based gas sensor were examined at one-minute intervals after exposing the device to NO_2_ gas with different concentrations (0 to 5 ppm) (Figure 2g). The gas concentration was precisely controlled using nitrogen as a carrier gas, diluted via a mass flow controller. As the NO_2_ gas concentration increased, a threshold voltage (*V*th) positive shift of the TFT occurred and a decrease in the drain current (*I*_D_) was induced [64,65]. When the proposed gas sensor detects gas, the adsorption of NO_2_ gas molecules on the surface and the desorption and recovery processes are crucial. In the device exposed to NO_2_ gas, the gas molecules were strongly bound to the surface due to the oxidized channel, and the electrical characteristics of the device were not recovered after N_2_ gas purging. In general, gas sensors operate on the principle of molecular desorption after the application of external energy, which recovers their electrical characteristics [66,67]. Therefore, we evaluated the recovery performance of the IGZO TFT-based NO_2_ gas sensor by applying light energy with light-emitting diodes of different wavelengths such as a UV light-emitting diode (LED) (400 nm), blue LED (450 nm), white LED, and red LED (635 nm) to the device exposed to NO_2_ gas (concentration: 5 ppm) (Figure 2h). The proposed devices exposed to NO_2_ gas recovered completely to the pristine state when light energy from UV LED, blue LED, and white LED lights was applied. The devices exposed to NO_2_ gas recovered completely to the pristine state when light energy from UV LED, blue LED, and white LED lights were applied. However, they did not recover when a red LED was applied, which means the absorbance spectrum of IGZO is almost zero in the red region, meaning that electron–hole pairs are not formed by the light, and therefore desorption of NO_2_ molecules cannot occur. More specifically, the injected NO_2_ molecules are adsorbed on the surface of IGZO channel (Figure 2i). The adsorbed NO_2_ molecules capture electron carriers in the IGZO channel, reducing the electron carrier’s concentration in the channel, thus causing the *V*th to shift positively and the *I*_D_ to decrease (Figure 2j). Finally, the electron–hole pairs generated by the light illumination desorb the NO_2_ molecules, and the *V*th and *I*_D_ recover to a pristine state, allowing for NO_2_ gas detection using the mechanism. A notable aspect of this gas sensor research effort is the successful initialization of the sensor by irradiating light sources beyond the bandgap at room temperature, instead of applying conventional thermal energy for the desorption of gas molecules necessary for sensor initialization. This approach eliminates the risk associated with the use of thermal energy when configuring sensors for hazardous gases, such as explosive gases like hydrogen or oxygen. Table 1 summarizes the performance of IGZO based gas sensors.

### 2.2. Logic Circuits Application

The NAND and NOR gates, fundamental building blocks of logic circuit systems, are recognized as universal gates, as all other types of gates can be constructed through combinations of these two. Consequently, extensive research efforts have been dedicated to developing more efficient NAND and NOR gates. In addition, ring oscillators serve as critical circuit components through their applications in clock generation, frequency synthesis, and random number generation. Beyond traditional Boolean logic applications, recent studies have proposed MVL as a promising approach to addressing various demands such as fundamental hardware architecture optimization, reduced power consumption, and circuit complexity. Unlike conventional binary logic, which relies solely on “0” and “1”, MVL employs three or more logic states, enhancing information storage density while minimizing the number of electronic components. For instance, MVL systems that incorporate ternary (0, 1, 2) or quaternary (0, 1, 2, 3) states can improve data density, reduce interconnect overhead, and enhance computational efficiency. The following studies highlight efforts to implement high-performance logic circuits using IGZO [71,72].

**Figure 3:**
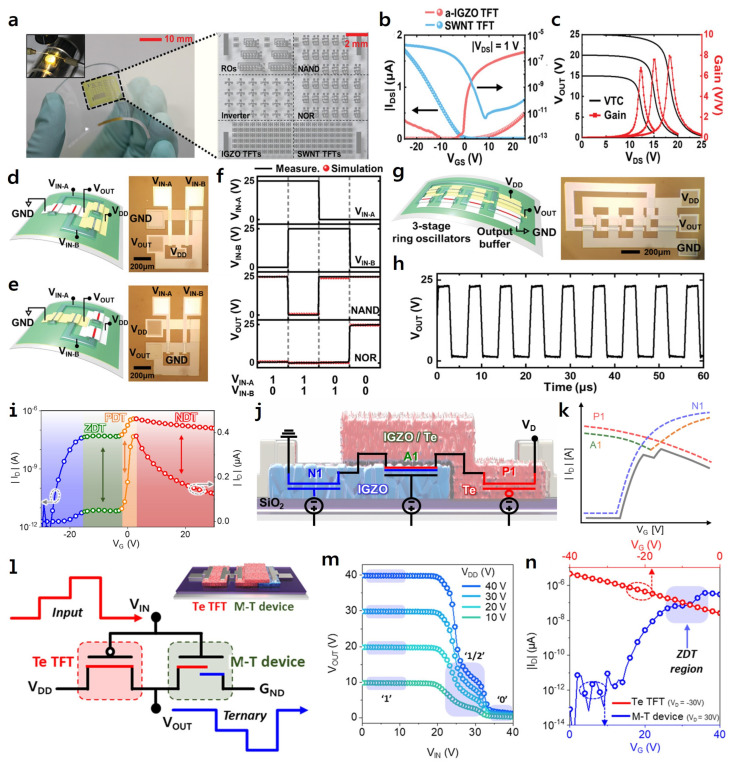
(**a**) An optical image of layered photosensitive complementary logic circuits on a PI substrate and optical image of the logic circuit demonstrated with IGZO and SWNT TFTs IGZO. (**b**) Transfer curves of IGZO and SWNT TFTs. (**c**) Complementary inverter characteristics for V_DD_ applied from 15 V to 25 V and voltage gain at V_DD_ = 25 V of the complementary inverter. Schematic diagram and an OM image of (**d**) NAND and (**e**) NOR logic circuit. (**f**) Input and output signals (V_IN-A_, V_IN-B_) based on IGZO and SWNT TFT-based NAND and NOR logic circuits. (**g**) Schematic diagram and optical microscope image of demonstrated ROs and (**h**) output signal from the ROs. Reproduced with permission. Ref. [32] Copyright 2021, Wiley-VCH GmbH. (Reprint by permission of John Wiley & Sons). (**i**) Transfer curve of the M-T device at different gate bias voltages. (**j**) Equivalent circuit model schematic diagram with the Te TFT, IGZO TFT, and Te/IGZO TFT components. (**k**) Schematic diagram for explaining the current characteristics of the M-T device. Complementary ternary logic circuit based on M-T device of (**l**) schematic circuit diagram and (**m**) VTC curve. (**n**) Transfer curve depicting the performance of the Te TFT and the M-T device in the ternary inverter. Reproduced with permission. Ref. [79] Copyright 2024, American Chemical Society.

Jeong et al. fabricated logic circuits such as complementary inverters, NAND, NOR, and ring oscillators (ROs) using components based on n-type IGZO and p-type SWNT IGZO on polyimide (PI) films (Figure 3a) [32]. First, to evaluate whether the pull-down and pull-up operations, which are fundamental for stable inverter operation with low leakage current, functioned effectively, the transfer curve of the SWNT and IGZO transistors were investigated (Figure 3b). The IGZO (or SWNT) transistors exhibited a high on/off ratio of ≈10^7^ A (≈10^5^ A) and negligible off-current (≈10 pA) at |*V*_DS_| = 1 V. Based on these electrical characteristics, a complementary inverter’s voltage transfer characteristic (VTC) curve demonstrated with IGZO TFT and SWNT TFT confirmed full-swing VTC operation with a voltage gain of eight (Figure 3c). Moreover, advanced complementary logic circuits, including NAND and NOR gates, were demonstrated, with both schematic representations and optical microscope (OM) images presented for the NAND and NOR gates constructed using IGZO/SWNT TFTs (Figure 3d,e). For the NAND gate demonstration [73,74], two IGZO TFTs were connected in series, and then two SWNT TFTs were connected in parallel. In the case of the NOR gate [75,76], two SWNT TFTs were arranged in series, and then two IGZO TFTs were configured in parallel. The fabricated logic circuits were operated by applying two input voltage signals (V_IN-A_ = 0 V and V_IN-B_ = 25 V) at different time intervals. Furthermore, in digital logic circuits, input and output voltages of 0 V and 25 V correspond to “0 states” and “1 states” (binary code), respectively. Therefore, two input signals generated combinations of digital input signals (i.e., 00, 01, 10, 11), enabling the successful demonstration of the NAND and NOR logic circuit (Figure 3f). Additionally, three-stage ROs with output buffers were also demonstrated, and the schematic and OM images of the fabricated Ros presented (Figure 3g). In the case of the ROs, three complementary inverters were arranged in series [77,78], causing the output voltage to oscillate, and the output buffer amplified the output signal and reduced external noise to stabilize the signal. Also, the fabricated ROs exhibited a voltage swing ranging from 1 V to 23 V. Furthermore, the oscillation frequency (*f*_osc_) was extracted as 142.9 kHz, and the stage delay was calculated as (2n*f*_osc_)^−1^, resulting in a stage delay of 0.87 µs, demonstrating the performance of the ROs.

In 2024, Lee et al. demonstrated a tellurium (Te) and IGZO-based partially overlapped heterojunction transistor and expand to ternary logic circuit [79]. The proposed mixed transconductance transistor (M-T device) sequentially exhibits unique current behaviors of zero differential transconductance (ZDT), positive differential transconductance (PDT), and negative differential transconductance (NDT) according to the gate voltage (*V_G_*) sweep on the transfer curve (Figure 3i). The inherent variable transconductance characteristics of the M-T device can be explained by analyzing the channel region by dividing it into three distinct segments. Figure 3j shows an equivalent circuit model explaining the unique behavior of the proposed M-T device, in which Te TFT (P1), Te/IGZO ambipolar TFT (N1), and IGZO TFT (P1) are interconnected in series. Therefore, the unique electrical characteristics of the M-T device can be explained by the variation of the transistor transconductance due to the combined resistance of each component interconnected in series (Figure 3k). For the application of MVL, a ternary inverter was demonstrated by connecting the proposed M-T device and the Te TFT (Figure 3l). The ternary inverter achieved three distinct logic states (V_DD_ = 1 state, V_DD/2_ = 1/2 state, G_ND_ = 0 state) on the VTC as shown in Figure 3m. The ternary inverter exhibited maximum gain of 15.3 V·V^−1^ (V_DD_ = 50 V), demonstrating effective demonstration of three logic states (Figure 3n). Matching the clear ternary operation to the ZDT region (27 V < *V_G_* < 32 V) of the M-T device and Te TFT has an impact on the implementation of the intermediate logic state. Table 2 summarizes the performance of IGZO based logic circuit.

### 2.3. IGZO-Based Biosensors

In modern society, health monitoring and management methods are gaining attention with the increasing attention on individual quality of life and the aging population. In particular, early diagnosis of diseases is important to maximize treatment for conditions such as diabetes, cardiovascular disease, and various cancers. Therefore, biosensors that convert biological signals into electrical signals to diagnose abnormal signs in the human body, and their application devices, are being actively studied. Recently, biosensors that collect amounts of human signals such as oxygen saturation [88,89], blood sugar [90,91], and heart rate [92,93] through integration with smart devices are under development for health diagnostics. In addition, research is being conducted on the integration of flexible components for smooth contact with the curved surface of human skin and real-time monitoring through mobile wearable devices [94,95,96]. In this way, next-generation biosensors demand key properties such as accuracy, selectivity, stability, limit of detection (LOD), and fast response.

**Figure 4:**
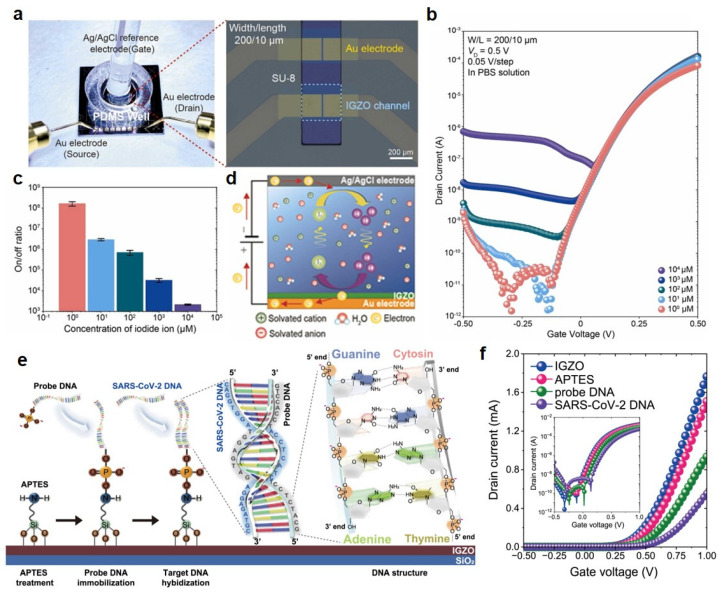
(**a**) Photograph and OM image of IGZO-based electrolyte-gated thin-film transistors (EGTFTs) with channel widths (200 µm) and length (10 µm). (**b**) Electrical characteristics of IGZO-EGTFT with different iodide concentration (1 to 10^4^ µM). (**c**) On/off-current ratio of IGZO-EGTFT measured at *V_G_* = 0.5, −0.3 V. (**d**) Schematic diagram of the charge transport mechanism with electrolyte-Ag/AgCl reference electrode (RE). Reproduced with permission. Ref. [97] Copyright 2021, Elsevier B.V. (**e**) Functionalization of the IGZO surface for SARS-CoV-2 DNA detection includes a several steps: APTES treatment, probe DNA immobilization, and SARS-CoV-2 DNA hybridization. (**f**) Transfer properties of IGZO-EGTFT with treatment of APTES, immobilization of probe DNA, and hybridization of SARS-CoV-2 DNA in PBS solution. Reproduced with permission. Ref. [33] Copyright 2024, Elsevier.

For sensing iodide ions, Park et al. demonstrated electrolyte-gated thin-film transistors (EGTFTs) using IGZO based on a sol–gel process [97]. Figure 4a shows a photograph and OM image of the IGZO-EGTFTs. An Ag/AgCl reference electrode for applying the gate bias voltage was placed in the electrolyte solution confined in a polydimethylsiloxane (PDMS) well. In addition, the whole area except for the IGZO channel region was passivated using SU-8 2000. Figure 4b exhibits the changed electrical properties dependent on the iodine ion concentration in the phosphate-buffered saline (PBS) solution. The concentration of the detected iodine ions was from 1 μM to 10^4^ μM, and the off-current in the transfer curve gradually increased as the ion concentration increased. In particular, the off-current of the proposed device at 1 μM iodine ions achieved 1.61 × 10^8^ and decreased to 2.14 × 10^3^ at 10^4^ μM. This indicates that the on/off ratio decreases as the concentration of iodide ions increases due to the increased off-current (Figure 4c). In addition, the calibration curve of the *I*_D_ at the optimized *V_G_* of −0.3 V has a high linear sensitivity with an R^2^ value of 0.992. Also, the electrical properties of the proposed biosensor are explained through the charge transfer behavior at the interface (Figure 4d). As the iodine ion concentration in the electrolyte solution increases, the redox ions diffuse at the IGZO and electrolyte interface, lowering the interfacial resistance and increasing the *I*_D_ in the transfer curve.

In 2024, Park et al. reported the demonstration of an IGZO-based EGTFT capable of detecting SARS-CoV-2. The proposed device sequentially combined organic materials on the surface of the deposited IGZO channel layer [33]. As shown in Figure 4e, (3-Aminopropyl)triethoxysilane (APTES) was functionalized on the surface of the IGZO layer. The silane group of APTES facilitates binding to the surface of IGZO and provides stable covalent bonding for deoxyribonucleic acid (DNA), antigens, antibodies, etc. Then, the probe DNA modified to be dependent on SARS-CoV-2 is covalently immobilized. At this time, a covalent bond is formed due to the stable interaction between the head group of APTES and the phosphate group of the coated probe DNA. Then, the probe DNA immobilizes the SARS-CoV-2 DNA. Figure 4f exhibits the transfer curve of the proposed device according to the chemical binding of each organic material. As the step-by-step process progresses in the transfer curve, the *V*th shifts in a positive direction, and the *I*_D_ decreases. This is because APTES achieves covalent bonding with the hydroxylated IGZO surface, while the phosphate backbone of DNA induces negative charge accumulation in the IGZO channel with the sequential immobilization of probe DNA and SARS-CoV-2 DNA. Table 3 summarizes the performance of IGZO based biosensor.

### 2.4. Neuromorphic Devices

In recent decades, the computer structure based on the architecture of von Neumann has become the basic standard of modern computers. However, in modern society, where high volumes of data are handled, the typical von Neumann computer system has a bottleneck phenomenon that hinders high-speed operation between the CPU and memory, and causes high power consumption during data processing [104,105]. To overcome these limitations of the von Neumann architecture, neuromorphic technology that mimics the human brain structure has emerged as a promising solution [106,107,108]. However, neuromorphic computing that imitates the human brain consumes about 20 W of power, enables parallel structure processing of data, can significantly alleviate the bottleneck phenomenon between memory and CPU, and achieve low power consumption [107,109,110]. In addition, it is considered a means of significantly improving mass data processing time and learning speed to advance artificial intelligence, autonomous driving, and deep learning technologies. The key point of neuromorphic computing is to imitate the functions of human neurons and synapses with electrical circuits [111,112]. The weight of the synapse is determined according to the occurrence time and interval of the spike signal during the signal transmission process between the presynaptic neuron and the postsynaptic neuron, and this mechanism is called spike-timing dependent plasticity (STDP) [113,114,115]. Therefore, neuromorphic devices perform inhibition and reinforcement of long-term and short-term memory through the STDP algorithm.

**Figure 5:**
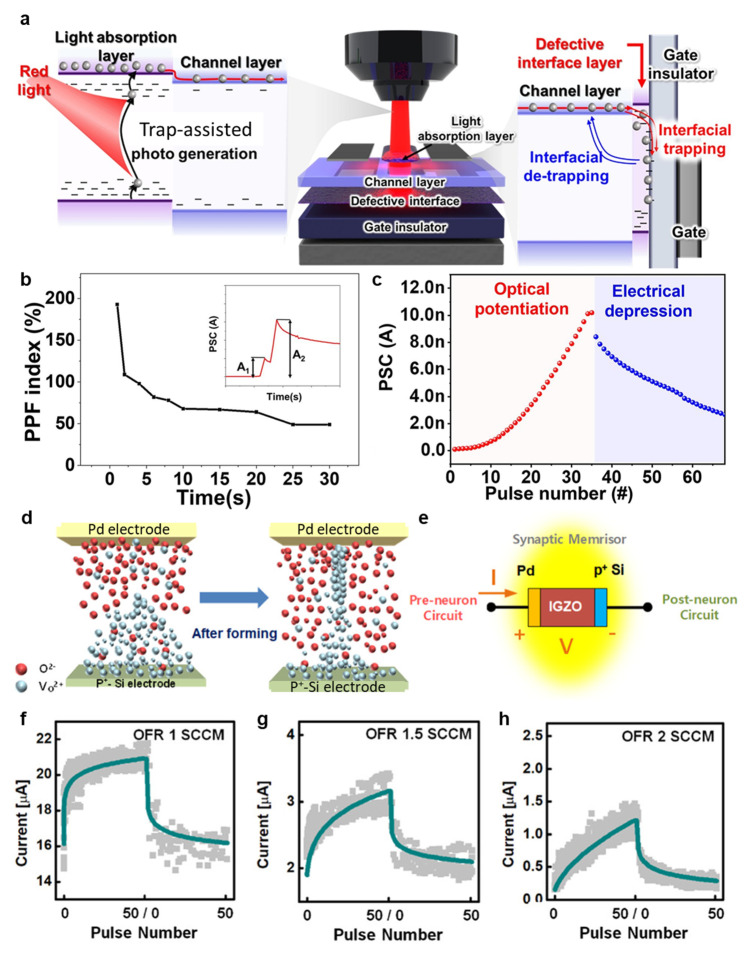
(**a**) Synaptic behavior mechanism of IGZO transistor with synaptic function. (**b**) The correlation between the PPF index and the interval time of the optoelectronic IGZO synaptic transistor with the defective interface layer. (**c**) Optical potentiation and electrical depression of optoelectronic IGZO synaptic transistor. Reproduced with permission. Ref. [116] Copyright 2022, Elsevier B.V. (**d**) Redistributions of O^2−^ and Vo^2+^ during the forming process. (**e**) Schematic of fabricated IGZO memristor-based synaptic device. Learning behaviors of IGZO memristor-based synaptic devices with switching layers prepared by OFRs of (**f**) 1 sccm, (**g**) 1.5 sccm, and (**h**) 2 sccm. Reproduced with permission. Ref. [117] Copyright 2020, American Chemical Society.

Kim et al. demonstrated a transistor that absorbs red light at 635 nm and performs synaptic functions by controlling defects in IGZO [116]. The proposed device consists of a double-layer IGZO structure. First, a defect interface layer created through sol–gel synthesis has a certain amount of surface dangling bonds and trap sites due to precursor decomposition and solvent evaporation. Then, a channel layer that controls the switching characteristics in response to light signals is continuously deposited using a sputtering method. The light-absorbing layer is annealed at 200 °C to expand the region to the visible light absorption band. Compared to the channel layer, the light-absorbing layer has enhanced oxygen defects due to increased V_O_ and –OH, creating sub-gap states that improve light absorption. Therefore, the transfer curve of the proposed device shows the conventional n-type transistor behavior, showing a negative shift in *V*th and an increase in photocurrent with red light irradiation. Figure 5a shows the band diagram and the synaptic operation mechanism of the proposed device. The carriers photogenerated by red light irradiation are trapped between the dielectric gate and the channel, which is a defective interface layer due to the gate bias. The defective interface layer increases the speed of trap and de-trap of the photogenerated carriers, enhancing the persistent photoconductance (PPC), and improving the postsynaptic current (PSC) and gain with the increased amount of photogenerated carriers in the light-absorbing layer. Figure 5b shows the paired pulse facilitation (PPF) index of the subsequent signal generated in the postsynaptic neuron by repeated stimulation in the proposed device. When the red light pulses with the shortest interval (t = 1 s) are irradiated, the PPF shows a maximum of 198% and decreases as the interval increases. Figure 5c shows the PSC characteristics in the proposed device, showing the photo-induced potential and electrical depression.

In 2020, Kim et al. developed a memristor-based synapse device consisting of a *p*^+^ Si/IGZO/Palladium (Pd) structure with two terminals [117]. The *p*^+^ Si is used as the bottom electrode (BE), and Pd is used as the top electrode (TE). Figure 5d presents the operating mechanism of the proposed device. The TE and BE of the proposed device have different oxygen contents. When a high negative voltage bias is applied to the Pd TE, O^2−^ ions from the IGZO move towards the *p*^+^ Si BE. At that time, Vo^2+^ ions migrate towards the TE to form conductive filaments due to the free energy of the Si-O bond being lower than that of the Pd-O bond. The schematic of a similar biological IGZO-based memristor device is shown in Figure 5e. For the learning operation in the proposed device, the *p*^+^ Si BE is grounded, and a voltage pulse is applied to the Pd TE. Figure 5f,g shows the learning behavior characteristics of the proposed device according to the oxide flow rate (OFR) during IGZO sputtering. Also, the inference current decreases with an increase in injected oxygen, reducing energy consumption. Table 4 summarizes the performance of IGZO based neuromorphic devices.

### 2.5. Photo Detectors

In a rapidly advancing technological environment, image sensors have established themselves as crucial components in various application fields such as smartphones, autonomous vehicles, and medical imaging technologies Also, high-resolution and dynamic-range imaging technologies are utilized in security systems for surveillance and monitoring, contributing to public safety. Due to the recent advancements in artificial intelligence (AI) and machine learning, the role of image sensors is becoming increasingly significant, linked to the importance of functions such as facial recognition and scene analysis. Therefore, to satisfy the performance requirements of the previously mentioned applications, research efforts have accelerated in the utilization of materials such as metal oxides [127,128,129], transition metal dichalcogenides [130,131,132], perovskites [133,134,135], and other organic materials [136,137,138] for the implementation of high-performance photodetectors. In particular, metal oxides exhibit high sensitivity to light, allowing for the effective detection of even the faintest optical signals. Moreover, several experimental methods such as varying the composition ratio of metal oxides or chemical doping with organic materials enable tuning the electrical properties to optimize the performance of photodetectors. These additional methods ensure strong environmental resilience, ensuring stable performance under changing temperature and humidity conditions.

**Figure 6:**
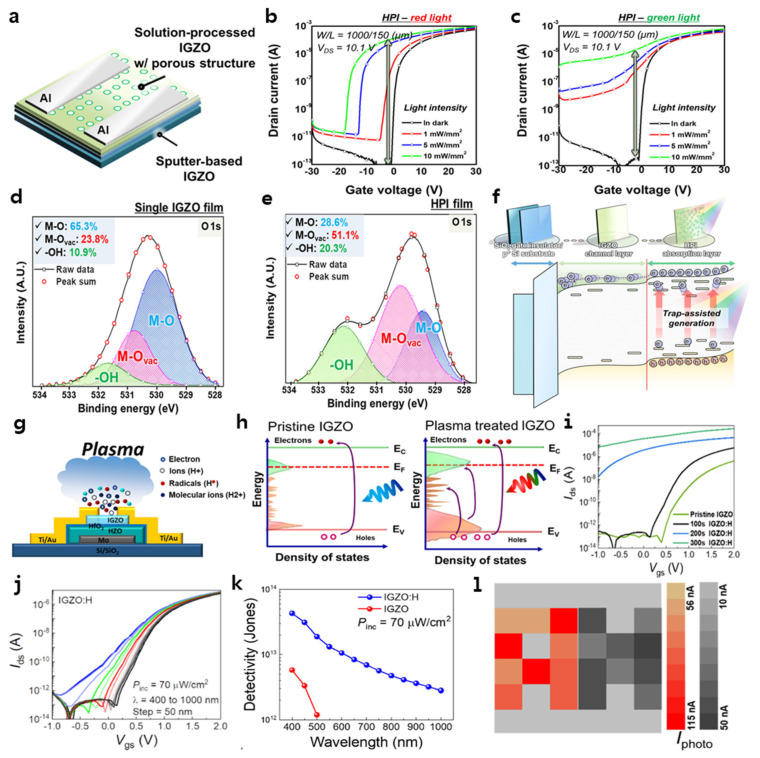
(**a**) Schematic of the device structure fabricated from homojunction-porous IGZO (HPI) thin films. Transfer characteristics of phototransistors based on HPI thin films when irradiated to (**b**) red light (635 nm) and (**c**) green light (532 nm). XPS results of (**d**) single IGZO and (**e**) HPI thin films with deconvoluted O 1s spectra. (**f**) Schematic of trap-assisted generation mechanism and electron transport in the IGZO phototransistor with the HPI absorption layer. Reproduced with permission. Ref. [139] Copyright 2021, American Chemical Society. (**g**) Schematic diagram of the photodetector and plasma processing. (**h**) Sub-gap state tuning and broad-spectrum detection mechanism enabled by plasma treatment. (**i**) Transfer curve of IGZO-based photodetectors depending on plasma treatment time. (**j**) The transfer curve of the IGZO photodetector exposed to the light of broad wavelengths. (**k**) Detectivity characteristics of IGZO and IGZO:H photodetectors investigated to evaluate the gas sensor performance induced by plasma treatment. (**l**) Photocurrent mapping of the IGZO:H photodetector array using 650 and 850 nm wavelengths. Reproduced with permission. Ref. [140] Copyright 2023, American Chemical Society.

Lee et al. demonstrated a homojunction-structured phototransistor with a sputtering-based IGZO channel and a solution-processed porous IGZO photosensitive layer (Figure 6a) [139]. The porosity of the IGZO photosensitive layer is attributed to the polyacrylate of the adhesive tape. Figure 6b,c shows the photo responses of the homojunction-porous IGZO (HPI) phototransistor to red (635 nm) and green (532 nm) light, respectively. The proposed HPI phototransistor exhibits a negative shift in *V*th and an increase in off-current after light irradiation, and the change becomes larger as the light intensity increases. In particular, under green light irradiation at 10 mW/mm^2^, it exhibits an on/off ratio of 4.92 × 10^2^ A/A, a *V*th of −6.27 V, and a photoresponsivity of 1.63 × 10^10^. X-ray photoelectron spectroscopy (XPS) analysis was used to examine the chemical composition of the IGZO film with and without porosity (Figure 6d,e). Compared to the single IGZO film, the HPI film shows an increase in peaks corresponding to oxygen vacancies (M−O_VAC_: 51.1%) and metal oxide–carbon groups by metal hydroxide or organic residues, respectively (-OH: 20.3%). This indicates an increase in trap sites due to impurities in the HPI film. As a result, the porosity of the HPI film enhances the photocurrent and light absorption efficiency. The physical defects of the porous film affect the photocatalytic activity and electron transport, with oxygen vacancies generated by the metal–oxygen defects at the pores considered as defect sites. In addition, the increased surface area due to the porosity enhances the light absorption and reactivity effect to increase the absorption efficiency. The increased oxygen vacancies generate numerous sub-gap states in the bandgap of the HPI phototransistor (Figure 6f). Based on UV−vis absorption and ultraviolet photoelectron spectroscopy (UPS) analysis, the bandgaps of the single IGZO and HPI films were extracted as 3.70 eV and 3.53 eV, respectively. Additionally, the HPI thin film has a lower valence band maximum (VBM: 2.85 eV) and conduction band minimum (CBM: 3.28 eV) compared to the single IGZO. Therefore, the bandgap alignment of the HPI phototransistor distinguishes the HPI thin film from the single IGZO layer, and the sub-gap states serve for electron–hole pair generation, contributing to photocurrent generation.

To enhance the light absorption efficiency of IGZO, Rho et al. performed H_2_ plasma treatment during an IGZO phototransistor fabrication process (Figure 6g) [140]. The light absorption of IGZO is confined to blue wavelengths (<500 nm), limiting its capacity to detect a broad range of the visible spectrum. However, when H_2_ plasma treatment is conducted on the IGZO surface, it reduces surface adsorbates and surface trap defect distribution (D_it_), forms −OH bonds at oxygen vacancy (V_o_) sites, and these −OH bonds along with hydrogen doping expand the sub-gap states near the VBM and CBM, increasing the carrier concentration. Therefore, H_2_ plasma treatment for IGZO enhances light absorption efficiency. This approach leverages the principle that, when low-energy photons are absorbed, photon-induced electrons are excited from the VBM to CBM through sub-gap states, producing a photocurrent (Figure 6h). To confirm the effect of H_2_ plasma treatment on IGZO TFTs, the electrical characteristics of these transistors were analyzed following treatment durations of 100 s, 200 s, and 300 s. The *I*_D_ shows a clear tendency to increase output current with longer plasma treatment times than the pristine IGZO TFT. Moreover, extended plasma treatment increases *I*_D_ and promotes a significant negative *V*th shift along with a higher subthreshold swing (SS), so the IGZO TFT treated with H_2_ plasma for 100 s shows the best electrical performance compared to the pristine state or the TFTs treated for 200 s and 300 s, resulting in its selection as the optimized condition for the phototransistor. Subsequently, the transfer curves of the IGZO TFT were examined under light exposure at wavelengths from 400 nm to 1000 nm to investigate their optical response characteristics (Figure 6j). Pristine IGZO demonstrates modulation of the *I*_D_ at λ = 400 nm, and minimal modulation at λ = 550 nm. However, H_2_ plasma-treated IGZO exhibits significant photocurrent across the wavelength spectrum from 400 nm to 1000 nm. IGZO An interesting aspect is that photocurrent is detected within the near-infrared region, which, as previously mentioned, is attributed to the sub-bandgap effects resulting from H_2_ plasma treatment.

Thus, IGZO demonstrates significant potential not only in the field of display technologies, but also across a range of next-generation advanced technological applications. However, despite the advantages offered by IGZO and other metal oxide semiconductors, critical material challenges remain in the realization of IGZO-based logic circuits and optical sensors. Chief among these is the absence of p-type metal oxide semiconductors necessary for the fabrication of complementary logic circuits [141]. While substantial efforts are underway to develop p-type semiconductors using copper oxide, such research remains in its early stages [142]. Additionally, hybrid semiconductor circuits incorporating organic p-type materials with inorganic oxides have been proposed as potential alternatives; however, the limited stability and low carrier mobility of organic materials continue to constrain the full potential of oxide semiconductors [143]. Another limitation is the excessively large bandgap of IGZO for use as a photosensor. With a bandgap of 3.70 eV, IGZO allows most visible light to pass through without absorption [79]. Consequently, utilizing IGZO as a photosensor necessitates the addition of layers that can absorb light at specific wavelengths and generate electron–hole pairs. However, this introduces a challenge, as carriers generated in the absorption layer must transfer to the IGZO channel layer, potentially leading to a loss in quantum efficiency. Therefore, the development of tailored materials that minimize quantum efficiency losses is essential for advancing the use of IGZO as a photosensor. We believe that addressing these challenges will establish metal oxide semiconductors, such as IGZO, as indispensable materials in the field of future application circuits. Table 5 summarizes the performance of IGZO based photo detectors.

## 3. Conclusions

In summary, this review paper comprehensively discusses various examples of IGZO applications. IGZO has been primarily used as a driving device for LCD and OLED in the display industry. However, its high charge carrier mobility and process compatibility have positioned it as a key material for next-generation computing devices. Based on these characteristics, IGZO can be applied to sensor technology, including gas sensors, biosensors, and photodetectors, utilizing its high sensitivity and stability. Furthermore, IGZO-based compact circuits enable high-density integration with low power consumption. Also, IGZO is gaining attention in the development of neuromorphic devices as a core material for next-generation computing technology that mimics neural networks. Thus, IGZO demonstrates application potential not only in the display field but also in advanced technology fields, and it is expected to play a pivotal role in the future of next-generation devices.

## Figures and Tables

**Table 1:** Summarization of IGZO-based gas sensor performance.

Device Type	Detect Gas	Detect Concentration	Responsivity	Stability	Sensing Condition	Ref.
Diode	C_2_H_5_OH	1000 ppm	186.1	N/A	250 °C	[25]
Transistor	NO_2_	5 ppm	500	40 days	RT	[29]
Diode	NH_3_	1000 ppm	≈1500	90 days	250 °C	[54]
Diode	NO_2_	25 ppm	≈110	60 days	250 °C	[55]
Diode	H_2_	100 ppm	482	30 days	RT	[56]
Diode	O_3_	5 ppm	74	60 days	RT	[57]
Diode	O_3_	5 ppm	62	90 days	RT	[63]
Transistor	NO_2_	40 ppm	≈10	N/A	RT	[68]
Diode	O_3_	5 ppm	45	1 day	RT	[69]
Diode	C_2_H_5_OH	1250 ppm	89.2	N/A	250 °C	[70]

**Table 2:** Summarization of IGZO-based logic circuit performance.

Pull−Up Device	Pull−Down Device	V_DD_	Gain (V/V)	Noise Margin	Additional Logic	Ref
SWNT	IGZO	25 V	≈8	N/A	NAND, NOR, XOR, XNOR, ROs	[32]
IGZO/Te	Te	50 V	24.7	N/A	Ternary	[79]
Resistor	IGZO	5 V	15	N/A	NAND, NOR	[80]
Resistor	IGZO	10 V	1939	≈8.7 V	N/A	[81]
Resistor	IGZO	2 V	163	N/A	ROs	[82]
SnO	IGZO	3 V	226	1.79 V	ROs	[83]
SnO_x_	IGZO	10 V	33.4	9.4 V	N/A	[84]
WSe_2_	IGZO	3 V	≈7	N/A	Rectifier	[85]
IGZO	IGZO	40 V	9.65	32.8 V	N/A	[86]
BTBT/DNTT	IGZO	20 V	≈60	15 V	N/A	[87]

**Table 3:** Summarization of IGZO-based biosensor circuit performance.

Sensing Device	Sensitivity (mV/pH)	V_H_ (mV)	R_D_ (mV/h)	V_H_ to Sensitivity (%)	R_D_ to Sensitivity (%)	Ref.
IGZO/SnO_2_	57.4	21.1	46.3	N/A	N/A	[26]
IGZO/SnO_2_	56.4	5.6	9.8	10.0	17.3	[98]
IGZO/SnO_2_	383	107	32.7	27.9	8.5	[99]
IGZO/IGZO	52.06	11	12.74	21.12	24.47	[100]
IGZO/SnO_2_	57.77	5.29	7.84	9.1	13.3	[101]
IGZO/SnO_2_	55.6	12	13.9	N/A	N/A	[102]
IGZO/SnO_2_	50.5	34.5	29	N/A	N/A	[103]

**Table 4:** Summarization of IGZO-based neuromorphic devices’ circuit performance.

Device Structure	PPF_max_	G_max_/G_min_	Recognition Rate (Accuracy)	Cycle	ΔT (s)	Ref.
LATP/IGZO	N/A	≈2	≈90%	50	0.001 s	[111]
IGZO	≈150%	17.4	≈90%	64	0.02 s	[118]
IGZO/SnO/SnS	≈112%	≈82	≈90%	40	5 s	[119]
IGZO/PVK/IGZO	≈300%	≈4.7	N/A	45	5 s	[120]
Al NPs/IGZO	≈160%	N/A	≈90%	100	0.1 s	[121]
Casein/IGZO	≈170%	≈3	90.5%	30	0.2 s	[122]
IGZO/Graphene oxide	182%	2466	73%	200	0.05 s	[123]
TaO_X_/Al_2_O_3_/IGZO	≈130%	≈2	95.1%	20	0.00001 s	[124]
TaO_X_/Al_2_O_3_/IGZO	N/A	137.2	98.08%	1000	0.001 s	[125]
TiO_2_/Al_2_O_3_/IGZO	≈150%	≈50	≈90%	200	0.1 s	[126]

**Table 5:** Summarization of IGZO-based photo detectors’ circuit performance.

Device Structure	Wavelength (nm)	Responsivity (A∙W^−1^)	Detectivity (Jones∙W^−1^)	Sensitivity	EQE (%)	Ref.
HZO/HfO_2_/IGZO	400–1000	≈2 × 10^4^	≈4 × 10^13^	≈10^4^	≈10^5^	[140]
IGZO/ZnON	405–635	≈1	2 × 10^14^	≈10^6^	≈10^4^	[144]
IGZO/Se	405–635	1.39 × 10^3^	3.44 × 10^13^	4.39 × 10^9^	3.52 × 10^9^	[145]
IGZO/BFP	365	44.8	1.3 × 10^15^	≈2 × 10^8^	≈103	[146]
IGZO/PbS QDs	1064	0.623	1.3 × 10^11^	≈8.85 × 10^7^	2248	[147]
PEDOT:PSS/ SnO_X_/IGZO	320–550	984	3.3 × 10^14^	≈10^6^	≈10^7^	[148]
IGZO	400–980	3 × 10^4^	5.3 × 10^17^	N/A	10^7^	[149]
Nanoporous IGZO	405–852	27.4	≈10^11^	1.1 × 10^4^	N/A	[150]
IGZO/BA_2_MA_3_Pb_4_I_13_	520	835.7	5.4 × 10^12^	≈10^5^	N/A	[151]
IGZO(PDI−BDT−O)	633	212	2 × 10^12^	N/A	N/A	[152]

## References

[1] Nomura K., Ohta H., Takagi A., Kamiya T., Hirano M., Hosono H. (2004). Room-temperature fabrication of transparent flexible thin-film transistors using amorphous oxide semiconductors. Nature.

[2] Shiah Y.-S., Sim K., Shi Y., Abe K., Ueda S., Sasase M., Kim J., Hosono H. (2021). Mobility–stability trade-off in oxide thin-film transistors. Nat. Electron..

[3] Sheng J., Hong T., Lee H.-M., Kim K., Sasase M., Kim J., Hosono H., Park J.-S. (2019). Amorphous IGZO TFT with high mobility of ∼70 cm^2^/(V s) via vertical dimension control using PEALD. ACS Appl. Mater. Interfaces.

[4] Park Y., Cho D.Y., Kim R., Kim K.H., Lee J.W., Lee D.H., Jeong S.I., Ahn N.R., Lee W., Choi J.B. (2022). Defect Engineering for High Performance and Extremely Reliable a-IGZO Thin-Film Transistor in QD-OLED. Adv. Electron. Mater..

[5] Kim D., Kim Y., Lee S., Kang M.S., Kim D.H., Lee H. (2017). High resolution a-IGZO TFT pixel circuit for compensating threshold voltage shifts and OLED degradations. IEEE J. Electron Devices Soc..

[6] Huang S., Jin J., Kim J., Wu W., Song A., Zhang J. (2023). IGZO Source-Gated Transistor for AMOLED Pixel Circuit. IEEE Trans. Electron Devices.

[7] Kang S.-H., Jo J.-W., Lee J.M., Moon S., Shin S.B., Choi S.B., Byeon D., Kim J., Kim M.-G., Kim Y.-H. (2024). Full integration of highly stretchable inorganic transistors and circuits within molecular-tailored elastic substrates on a large scale. Nat. Commun..

[8] Geng D., Wang K., Li L., Myny K., Nathan A., Jang J., Kuo Y., Liu M. (2023). Thin-film transistors for large-area electronics. Nat. Electron..

[9] Oh H., Oh J.-Y., Park C.W., Pi J.-E., Yang J.-H., Hwang C.-S. (2022). High density integration of stretchable inorganic thin film transistors with excellent performance and reliability. Nat. Commun..

[10] Cho M.H., Choi C.H., Seul H.J., Cho H.C., Jeong J.K. (2021). Achieving a low-voltage, high-mobility IGZO transistor through an ALD-derived bilayer channel and a hafnia-based gate dielectric stack. ACS Appl. Mater. Interfaces.

[11] Kim D.-G., Ryu S.-H., Jeong H.-J., Park J.-S. (2021). Facile and stable n^+^ doping process via simultaneous ultraviolet and thermal energy for coplanar ALD-IGZO thin-film transistors. ACS Appl. Electron. Mater..

[12] Kim J., Park J., Yoon G., Khushabu A., Kim J.-S., Pae S., Cho E.-C., Yi J. (2020). Effect of IGZO thin films fabricated by Pulsed-DC and RF sputtering on TFT characteristics. Mater. Sci. Semicond. Process..

[13] Guo M., Ou H., Xie D., Zhu Q., Wang M., Liang L., Liu F., Ning C., Cao H., Yuan G. (2023). Critical assessment of the high carrier mobility of bilayer In2O3/IGZO transistors and the underlying mechanisms. Adv. Electron. Mater..

[14] Wen P., Peng C., Chen Z., Ding X., Chen F.-H., Yan G., Xu L., Wang D., Sun X., Chen L. (2024). High mobility of IGO/IGZO double-channel thin-film transistors by atomic layer deposition. Appl. Phys. Lett..

[15] Wu H.-C., Chien C.-H. (2014). Highly transparent, high-performance IGZO-TFTs using the selective formation of IGZO source and drain electrodes. IEEE Electron Device Lett..

[16] Lee S.H., Lee S., Jang S.C., On N., Kim H.-S., Jeong J.K. (2021). Mobility enhancement of indium-gallium oxide via oxygen diffusion induced by a metal catalytic layer. J. Alloys Compd..

[17] Jeong K., Shin D.Y., Park J., Yi D., Hong H., Kim H., Chung K. (2024). Noncontact Monitoring and Imaging of the Operation and Performance of Thin-Film Field-Effect Transistors. Adv. Sci..

[18] Liu K., Ouyang B., Guo X., Guo Y., Liu Y. (2022). Advances in flexible organic field-effect transistors and their applications for flexible electronics. npj Flex. Electron..

[19] Lee G., Jang S.C., Lee J.H., Park J., Noh B., Choi H., Kweon H., Kim D.H., Kim H.Y., Kim H. (2024). Intrinsically Photopatternable High-k Polymer Dielectric for Flexible Electronics. Adv. Funct. Mater..

[20] Kim J., Jang S.C., Bae K., Park J., Kim H.-D., Lahann J., Kim H.-S., Lee K.J. (2021). Chemically tunable organic dielectric layer on an oxide TFT: Poly(*p*-xylylene) derivatives. ACS Appl. Mater. Interfaces.

[21] Fu C., Li Z.-Y., Li Y.-J., Zhu M.-M., Luo L.-B., Jiang S.-S., Wang Y., Wang W.-H., He G. (2024). High-performance IGZO/In_2_O_3_ NW/IGZO phototransistor with heterojunctions architecture for image processing and neuromorphic computing. J. Mater. Sci. Technol..

[22] Zhu Y., Peng B., Zhu L., Chen C., Wang X., Mao H., Zhu Y., Fu C., Ke S., Wan C. (2022). IGZO nanofiber photoelectric neuromorphic transistors with indium ratio tuned synaptic plasticity. Appl. Phys. Lett..

[23] Liu J., Tang W., Liu Y., Yang H., Li X. Almost-nonvolatile IGZO-TFT-based near-sensor in-memory computing. Proceedings of the 2021 IEEE International Symposium on Circuits and Systems (ISCAS).

[24] Du Y., Tang J., Li Y., Xi Y., Li Y., Li J., Huang H., Qin Q., Zhang Q., Gao B. (2024). Monolithic 3D integration of analog RRAM-based computing-in-memory and sensor for energy-efficient near-sensor computing. Adv. Mater..

[25] Peng R.-Y., Liu W.-C. (2020). Study of a high-performance chemoresistive ethanol gas sensor synthesized with au nanoparticles and an amorphous IGZO thin film. IEEE Trans. Electron Devices.

[26] Pyo J.-Y., Cho W.-J. (2018). In-plane-gate a-IGZO thin-film transistor for high-sensitivity pH sensor applications. Sens. Actuators B Chem..

[27] Smith J.T., Shah S.S., Goryll M., Stowell J.R., Allee D.R. (2013). Flexible ISFET biosensor using IGZO metal oxide TFTs and an ITO sensing layer. IEEE Sens. J..

[28] Yang T.-H., Chen T.-Y., Wu N.-T., Chen Y.-T., Huang J.-J. (2017). IGZO-TFT biosensors for Epstein–Barr virus protein detection. IEEE Trans. Electron Devices.

[29] Vijjapu M.T., Surya S.G., Yuvaraja S., Zhang X., Alshareef H.N., Salama K.N. (2020). Fully integrated indium gallium zinc oxide NO_2_ gas detector. ACS Sens..

[30] Shin W., Kwon D., Ryu M., Kwon J., Hong S., Jeong Y., Jung G., Park J., Kim D., Lee J.-H. (2021). Effects of IGZO film thickness on H_2_S gas sensing performance: Response, excessive recovery, low-frequency noise, and signal-to-noise ratio. Sens. Actuators B Chem..

[31] Han K.-L., Lee W.-B., Kim Y.-D., Kim J.-H., Choi B.-D., Park J.-S. (2021). Mechanical durability of flexible/stretchable a-IGZO TFTs on PI island for wearable electronic application. ACS Appl. Electron. Mater..

[32] Jeong J., Seo S.G., Yu S., Kang Y., Song J., Jin S.H. (2021). Flexible Light-to-Frequency Conversion Circuits Built with Si-Based Frequency-to-Digital Converters via Complementary Photosensitive Ring Oscillators with p-Type SWNT and n-Type a-IGZO Thin Film Transistors. Small.

[33] Hwang C., Baek S., Song Y., Lee W.-J., Park S. (2024). Wide-range and selective detection of SARS-CoV-2 DNA via surface modification of electrolyte-gated IGZO thin-film transistors. Iscience.

[34] Subramanian Periyal S., Jagadeeswararao M., Ng S.E., John R.A., Mathews N. (2020). Halide perovskite quantum dots photosensitized-amorphous oxide transistors for multimodal synapses. Adv. Mater. Technol..

[35] Yoon J., Bae G.-Y., Yoo S., Yoo J.I., You N.-H., Hong W.-K., Ko H.C. (2020). Deep-ultraviolet sensing characteristics of transparent and flexible IGZO thin film transistors. J. Alloys Compd..

[36] Kim W.-G., Tak Y.J., Yoo H., Kim H.T., Park J.W., Choi D.H., Kim H.J. (2021). Photo-induced Reactive Oxygen Species Activation for Amorphous Indium–Gallium–Zinc Oxide Thin-Film Transistors Using Sodium Hypochlorite. ACS Appl. Mater. Interfaces.

[37] Hu C., Yu X., Li Y., Cheng J., Li Q., Xiao B. (2022). Bandgap engineering of strained S-terminated MXene and its promising application as NO_x_ gas sensor. Appl. Surf. Sci..

[38] Reddeppa M., Park B.-G., Murali G., Choi S.H., Chinh N.D., Kim D., Yang W., Kim M.-D. (2020). NO_x_ gas sensors based on layer-transferred n-MoS_2_/p-GaN heterojunction at room temperature: Study of UV light illuminations and humidity. Sens. Actuators B Chem..

[39] Sun B., Qin F., Jiang L., Gao J., Liu Z., Wang J., Zhang Y., Fan J., Kan K., Shi K. (2022). Room-temperature gas sensors based on three-dimensional Co_3_O_4_/Al_2_O_3_@Ti_3_C_2_T_x_ MXene nanocomposite for highly sensitive NO_x_ detection. Sens. Actuators B Chem..

[40] Huang C.-Y., He X.-R., Dai T.-Y. (2022). Realization of a self-powered Cu_2_O ozone gas sensor through the lateral photovoltaic effect. J. Mater. Chem. C.

[41] Russ T., Hu Z., Li L., Zhou L., Liu H., Weimar U., Barsan N. (2022). In operando investigation of the concentration dependent NO_2_ sensing mechanism of Bi_2_S_3_ nanorods at low temperatures and the interference of O_3_. ACS Sens..

[42] Han P., Mei H., Liu D., Zeng N., Tang X., Wang Y., Pan Y. (2021). Calibrations of low-cost air pollution monitoring sensors for CO, NO_2_, O_3_, and SO_2_. Sensors.

[43] Goswami P., Gupta G. (2022). Recent progress of flexible NO_2_ and NH_3_ gas sensors based on transition metal dichalcogenides for room temperature sensing. Mater. Today Chem..

[44] Nakarungsee P., Srirattanapibul S., Issro C., Tang I.-M., Thongmee S. (2020). High performance Cr doped ZnO by UV for NH_3_ gas sensor. Sens. Actuators A Phys..

[45] Fernández-Ramos M.D., Capitán-Vallvey L.F., Pastrana-Martínez L.M., Morales-Torres S., Maldonado-Hódar F.J. (2022). Chemoresistive NH_3_ gas sensor at room temperature based on the carbon gel-TiO_2_ nanocomposites. Sens. Actuators B Chem..

[46] Zhou L., Kato F., Nakamura N., Oshikane Y., Nagakubo A., Ogi H. (2021). MEMS hydrogen gas sensor with wireless quartz crystal resonator. Sens. Actuators B Chem..

[47] Singh A., Sikarwar S., Verma A., Yadav B.C. (2021). The recent development of metal oxide heterostructures based gas sensor, their future opportunities and challenges: A review. Sens. Actuators A Phys..

[48] Naqi M., Jang S.C., Cho Y., Park J.M., Oh J.O., Rho H.Y., Kim H.-S., Kim S. (2023). Low temperature processed, highly stable CMOS inverter by integrating Zn-ON and tellurium thin-film transistors. J. Inf. Disp..

[49] Nikolic M.V., Milovanovic V., Vasiljevic Z.Z., Stamenkovic Z. (2020). Semiconductor gas sensors: Materials, technology, design, and application. Sensors.

[50] Uma S., Shobana M.K. (2023). Metal oxide semiconductor gas sensors in clinical diagnosis and environmental monitoring. Sens. Actuators A Phys..

[51] Yamazoe N., Shimanoe K. (2020). Fundamentals of semiconductor gas sensors. Semiconductor Gas Sensors.

[52] Das S., Mojumder S., Saha D., Pal M. (2022). Influence of major parameters on the sensing mechanism of semiconductor metal oxide based chemiresistive gas sensors: A review focused on personalized healthcare. Sens. Actuators B Chem..

[53] Yang X., Deng Y., Yang H., Liao Y., Cheng X., Zou Y., Wu L., Deng Y. (2023). Functionalization of mesoporous semiconductor metal oxides for gas sensing: Recent advances and emerging challenges. Adv. Sci..

[54] Chen P.-L., Liu I.-P., Chen W.-C., Niu J.-S., Liu W.-C. (2020). Study of a platinum nanoparticle (Pt NP)/amorphous In-Ga-Zn-O (A-IGZO) thin-film-based ammonia gas sensor. Sens. Actuators B Chem..

[55] Eadi S.B., Shin H., Nguyen K.T., Song K.-W., Choi H.-W., Kim S.-H., Lee H.-D. (2022). Indium-gallium–zinc oxide (IGZO) thin-film gas sensors prepared via post-deposition high-pressure annealing for NO_2_ detection. Sens. Actuators B Chem..

[56] Huang W.-C., Li Y., Chang N.-H., Hong W.-J., Wu S.-Y., Liao S.-Y., Hsueh W.-J., Wang C.-M., Huang C.-Y. (2024). Highly stable and selective H_2_ gas sensors based on light-activated a-IGZO thin films with ZIF-8 selective membranes. Sens. Actuators B Chem..

[57] Huang C.-Y., He X.-R., Huang C.-T. (2022). Realization of a self-powered InGaZnO MSM ozone sensor via a surface state modulated photovoltaic effect. ACS Appl. Electron. Mater..

[58] Pham T., Li G., Bekyarova E., Itkis M.E., Mulchandani A. (2019). MoS_2_-based optoelectronic gas sensor with sub-parts-per-billion limit of NO_2_ gas detection. ACS Nano.

[59] Li G., Sun Z., Zhang D., Xu Q., Meng L., Qin Y. (2019). Mechanism of sensitivity enhancement of a ZnO nanofilm gas sensor by UV light illumination. ACS Sens..

[60] Li W., Guo J., Cai L., Qi W., Sun Y., Xu J.-L., Sun M., Zhu H., Xiang L., Xie D. (2019). UV light irradiation enhanced gas sensor selectivity of NO_2_ and SO_2_ using rGO functionalized with hollow SnO_2_ nanofibers. Sens. Actuators B Chem..

[61] Kaur N., Zappa D., Poli N., Comini E. (2019). Integration of VLS-Grown WO_3_ Nanowires into Sensing Devices for the Detection of H_2_S and O_3_. ACS Omega.

[62] Sui N., Wei X., Cao S., Zhang P., Zhou T., Zhang T. (2022). Nanoscale bimetallic AuPt-functionalized metal oxide chemiresistors: Ppb-level and selective detection for ozone and acetone. ACS Sens..

[63] Huang C.-Y., Juan K.-Y., Guo P.-H., Wu Y.-R., Kao S.-F., Liao S.-Y. (2024). Self-Powered InGaZnO Ozone Gas Sensors Based on a Metal–Semiconductor–Metal Structure with Asymmetric Interdigitated Electrodes. ACS Appl. Electron. Mater..

[64] Tabata H., Matsuyama H., Goto T., Kubo O., Katayama M. (2021). Visible-light-activated response originating from carrier-mobility modulation of NO_2_ gas sensors based on MoS_2_ monolayers. ACS Nano.

[65] Niu Y., Zeng J., Liu X., Li J., Wang Q., Li H., Rooij N.F.d., Wang Y., Zhou G. (2021). A photovoltaic self-powered gas sensor based on all-dry transferred MoS_2_/GaSe heterojunction for ppb-level NO_2_ sensing at room temperature. Adv. Sci..

[66] Wang B., Thukral A., Xie Z., Liu L., Zhang X., Huang W., Yu X., Yu C., Marks T.J., Facchetti A. (2020). Flexible and stretchable metal oxide nanofiber networks for multimodal and monolithically integrated wearable electronics. Nat. Commun..

[67] Vijjapu M.T., Surya S., Zalte M., Yuvaraja S., Baghini M.S., Salama K.N. (2021). Towards a low cost fully integrated IGZO TFT NO_2_ detection and quantification: A solution-processed approach. Sens. Actuators B Chem..

[68] Jang Y.-W., Kang J., Jo J.-W., Kim Y.-H., Kim J., Park S.K. (2021). Improved dynamic responses of room-temperature operable field-effect-transistor gas sensors enabled by programmable multi-spectral ultraviolet illumination. Sens. Actuators B Chem..

[69] Wu C.-H., Jiang G.-J., Chang K.-W., Lin C.-W., Chen K.-L. (2015). Highly sensitive amorphous In–Ga–Zn–O films for ppb-level ozone sensing: Effects of deposition temperature. Sens. Actuators B Chem..

[70] Chen H., Jiang W., Zhu L., Yao Y. (2017). Amorphous In–Ga–Zn–O powder with high gas selectivity towards wide range concentration of C_2_H_5_OH. Sensors.

[71] Cadilha Marques G., Weller D., Erozan A.T., Feng X., Tahoori M., Aghassi-Hagmann J. (2019). Progress report on “from printed electrolyte-gated metal-oxide devices to circuits”. Adv. Mater..

[72] Jeong J.W., Choi Y.-E., Kim W.-S., Park J.-H., Kim S., Shin S., Lee K., Chang J., Kim S.-J., Kim K.R. (2019). Tunnelling-based ternary metal–oxide–semiconductor technology. Nat. Electron..

[73] Park S., Lee H.J., Choi W., Jin H., Cho H., Jeong Y., Lee S., Kim K., Im S. (2022). Quaternary NAND logic and complementary ternary inverter with p-MoTe_2_/n-MoS_2_ heterostack channel transistors. Adv. Funct. Mater..

[74] Zheng Z., Zhang L., Song W., Feng S., Xu H., Sun J., Yang S., Chen T., Wei J., Chen K.J. (2021). Gallium nitride-based complementary logic integrated circuits. Nat. Electron..

[75] Chen H., Xue X., Liu C., Fang J., Wang Z., Wang J., Zhang D.W., Hu W., Zhou P. (2021). Logic gates based on neuristors made from two-dimensional materials. Nat. Electron..

[76] Migliato Marega G., Zhao Y., Avsar A., Wang Z., Tripathi M., Radenovic A., Kis A. (2020). Logic-in-memory based on an atomically thin semiconductor. Nature.

[77] Guo E., Xing S., Dollinger F., Hübner R., Wang S.-J., Wu Z., Leo K., Kleemann H. (2021). Integrated complementary inverters and ring oscillators based on vertical-channel dual-base organic thin-film transistors. Nat. Electron..

[78] Lei T., Shao L.-L., Zheng Y.-Q., Pitner G., Fang G., Zhu C., Li S., Beausoleil R., Wong H.-S.P., Huang T.-C. (2019). Low-voltage high-performance flexible digital and analog circuits based on ultrahigh-purity semiconducting carbon nanotubes. Nat. Commun..

[79] Lee D.H., Kim S., Woo G., Kim T., Kim Y.J., Yoo H. (2024). A Mixture of Negative-, Zero-, and Positive-Differential Transconductance Switching from Tellurium/Indium Gallium Zinc Oxide Heterostructures. ACS Appl. Mater. Interfaces.

[80] Naqi M., Cho Y., Kim S. (2023). High-speed current switching of inverted-staggered bottom-gate a-IGZO-based thin-film transistors with highly stable logic circuit operations. ACS Appl. Electron. Mater..

[81] Zhang Y., Lin Y., He G., Ge B., Liu W. (2020). Balanced performance improvement of a-InGaZnO thin-film transistors using ALD-derived Al_2_O_3_-passivated high-k HfGdOx dielectrics. ACS Appl. Electron. Mater..

[82] Pradhan J.R., Singh M., Dasgupta S. (2022). Inkjet-Printed, Deep Subthreshold Operated Pseudo-CMOS Inverters with High Voltage Gain and Low Power Consumption. Adv. Electron. Mater..

[83] Yuan Y., Yang J., Hu Z., Li Y., Du L., Wang Y., Zhou L., Wang Q., Song A., Xin Q. (2018). Oxide-based complementary inverters with high gain and nanowatt power consumption. IEEE Electron Device Lett..

[84] Yatsu K., Lee H.-A., Kim D.H., Park I.-J., Kwon H.-I. (2022). Highly stable oxide thin-film transistor-based complementary logic circuits under X-ray irradiation. ACS Appl. Electron. Mater..

[85] Lee S., Lee H.S., Yu S., Park J.H., Bae H., Im S. (2020). Tungsten Dichalcogenide Nanoflake/InGaZnO Thin-Film Heterojunction for Photodetector, Inverter, and AC Rectifier Circuits. Adv. Electron. Mater..

[86] Kim W., Lee W., Kwak T., Baek S., Lee S., Park S. (2022). Influence of UV/Ozone Treatment on Threshold Voltage Modulation in Sol–Gel IGZO Thin-Film Transistors. Adv. Mater. Interfaces.

[87] Han Y., Kim S., Kim C.-H., Yoo H. (2023). Experimental and theoretical evidence of charge injection barrier control by small-molecular charge injection layer and its effects on organic–Inorganic complementary inverters. IEEE Trans. Electron Devices.

[88] Sonmezoglu S., Fineman J.R., Maltepe E., Maharbiz M.M. (2021). Monitoring deep-tissue oxygenation with a millimeter-scale ultrasonic implant. Nat. Biotechnol..

[89] Chung H.U., Rwei A.Y., Hourlier-Fargette A., Xu S., Lee K., Dunne E.C., Xie Z., Liu C., Carlini A., Kim D.H. (2020). Skin-interfaced biosensors for advanced wireless physiological monitoring in neonatal and pediatric intensive-care units. Nat. Med..

[90] Srichan C., Srichan W., Danvirutai P., Ritsongmuang C., Sharma A., Anutrakulchai S. (2022). Non-invasively accuracy enhanced blood glucose sensor using shallow dense neural networks with NIR monitoring and medical features. Sci. Rep..

[91] Lipani L., Dupont B.G.R., Doungmene F., Marken F., Tyrrell R.M., Guy R.H., Ilie A. (2018). Non-invasive, transdermal, path-selective and specific glucose monitoring via a graphene-based platform. Nat. Nanotechnol..

[92] Kaisti M., Panula T., Leppänen J., Punkkinen R., Jafari Tadi M., Vasankari T., Jaakkola S., Kiviniemi T., Airaksinen J., Kostiainen P. (2019). Clinical assessment of a non-invasive wearable MEMS pressure sensor array for monitoring of arterial pulse waveform, heart rate and detection of atrial fibrillation. npj Digit. Med..

[93] Lee Y., Park J.-Y., Choi Y.-W., Park H.-K., Cho S.-H., Cho S.H., Lim Y.-H. (2018). A novel non-contact heart rate monitor using impulse-radio ultra-wideband (IR-UWB) radar technology. Sci. Rep..

[94] Ates H.C., Nguyen P.Q., Gonzalez-Macia L., Morales-Narváez E., Güder F., Collins J.J., Dincer C. (2022). End-to-end design of wearable sensors. Nat. Rev. Mater..

[95] Yang Y., Song Y., Bo X., Min J., Pak O.S., Zhu L., Wang M., Tu J., Kogan A., Zhang H. (2020). A laser-engraved wearable sensor for sensitive detection of uric acid and tyrosine in sweat. Nat. Biotechnol..

[96] Kim J., Campbell A.S., de Ávila B.E.-F., Wang J. (2019). Wearable biosensors for healthcare monitoring. Nat. Biotechnol..

[97] Hwang C., Kwak T., Kim C.-H., Kim J.H., Park S. (2022). Quantitative and rapid detection of iodide ion via electrolyte-gated IGZO thin-film transistors. Sens. Actuators B Chem..

[98] Hyun T.-H., Cho W.-J. (2023). High-Performance Potassium-Selective Biosensor Platform Based on Resistive Coupling of a-IGZO Coplanar-Gate Thin-Film Transistor. Int. J. Mol. Sci..

[99] Hong E.-K., Cho W.-J. (2021). High sensitivity In-Ga-Zn-O nanofiber-based double gate field effect transistors for chemical sensing. Sens. Actuators B Chem..

[100] Son H.W., Park J.H., Chae M.-S., Kim B.-H., Kim T.G. (2020). Bilayer indium gallium zinc oxide electrolyte-gated field-effect transistor for biosensor platform with high reliability. Sens. Actuators B Chem..

[101] Hyun T.-H., Cho W.-J. (2023). Fully transparent and highly sensitive pH sensor based on an a-IGZO thin-film transistor with coplanar dual-gate on flexible polyimide substrates. Chemosensors.

[102] Cho S.-K., Cho W.-J. (2021). Highly sensitive and transparent urea-EnFET based point-of-care diagnostic test sensor with a triple-gate a-IGZO TFT. Sensors.

[103] Jeon H.-U., Cho W.-J. (2021). Fully transparent and sensitivity-programmable amorphous indium-gallium-zinc-oxide thin-film transistor-based biosensor platforms with resistive switching memories. Sensors.

[104] Yang Y., Ostrowski D.P., France R.M., Zhu K., Van De Lagemaat J., Luther J.M., Beard M.C. (2016). Observation of a hot-phonon bottleneck in lead-iodide perovskites. Nat. Photonics.

[105] Yang J., Wen X., Xia H., Sheng R., Ma Q., Kim J., Tapping P., Harada T., Kee T.W., Huang F. (2017). Acoustic-optical phonon up-conversion and hot-phonon bottleneck in lead-halide perovskites. Nat. Commun..

[106] Wang C., Liu Y., Guo Y. (2024). Intrinsically flexible organic phototransistors for bioinspired neuromorphic sensory system. Wearable Electron..

[107] Markovic D., Mizrahi A., Querlioz D., Grollier J. (2020). Physics for neuromorphic computing. Nat. Rev. Phys..

[108] Song K.M., Jeong J.-S., Pan B., Zhang X., Xia J., Cha S., Park T.-E., Kim K., Finizio S., Raabe J. (2020). Skyrmion-based artificial synapses for neuromorphic computing. Nat. Electron..

[109] Wang T., Meng J., Zhou X., Liu Y., He Z., Han Q., Li Q., Yu J., Li Z., Liu Y. (2022). Reconfigurable neuromorphic memristor network for ultralow-power smart textile electronics. Nat. Commun..

[110] Göltz J., Kriener L., Baumbach A., Billaudelle S., Breitwieser O., Cramer B., Dold D., Kungl A.F., Senn W., Schemmel J. (2021). Fast and energy-efficient neuromorphic deep learning with first-spike times. Nat. Mach. Intell..

[111] Park J.-M., Hwang H., Song M.S., Jang S.C., Kim J.H., Kim H., Kim H.-S. (2023). All-solid-state synaptic transistors with lithium-ion-based electrolytes for linear weight mapping and update in neuromorphic computing systems. ACS Appl. Mater. Interfaces.

[112] Song Y.-W., Chang Y.-H., Choi J., Song M.-K., Yoon J.H., Lee S., Jung S.-Y., Ham W., Park J.-M., Kim H.-S. (2023). Doping modulated ion hopping in tantalum oxide based resistive switching memory for linear and stable switching dynamics. Appl. Surf. Sci..

[113] Prezioso M., Mahmoodi M.R., Bayat F.M., Nili H., Kim H., Vincent A., Strukov D.B. (2018). Spike-timing-dependent plasticity learning of coincidence detection with passively integrated memristive circuits. Nat. Commun..

[114] Wang Z., Zeng T., Ren Y., Lin Y., Xu H., Zhao X., Liu Y., Ielmini D. (2020). Toward a generalized Bienenstock-Cooper-Munro rule for spatiotemporal learning via triplet-STDP in memristive devices. Nat. Commun..

[115] Boyn S., Grollier J., Lecerf G., Xu B., Locatelli N., Fusil S., Girod S., Carrétéro C., Garcia K., Xavier S. (2017). Learning through ferroelectric domain dynamics in solid-state synapses. Nat. Commun..

[116] Chung J., Park K., Kim G.I., An J.B., Jung S., Choi D.H., Kim H.J. (2023). Visible light-driven indium-gallium-zinc-oxide optoelectronic synaptic transistor with defect engineering for neuromorphic computing system and artificial intelligence. Appl. Surf. Sci..

[117] Kim D., Jang J.T., Yu E., Park J., Min J., Kim D.M., Choi S.-J., Mo H.-S., Cho S., Roy K. (2020). Pd/IGZo/p^+^-Si synaptic device with self-graded oxygen concentrations for highly linear weight adjustability and improved energy efficiency. ACS Appl. Electron. Mater..

[118] Yang C.-H., Huang Y.-C., Shih L.-C., Mao S.-C., Wu J.-J., Chen J.-S. (2024). Harness Background Lighting for Separability Enhancement in Optical Sensing Reservoir for Temporal Signal Processing Utilizing Amorphous IGZO Neuromorphic Transistors. ACS Photonics.

[119] Zhang T., Fan C., Hu L., Zhuge F., Pan X., Ye Z. (2024). A Reconfigurable All-Optical-Controlled Synaptic Device for Neuromorphic Computing Applications. ACS Nano.

[120] Duan H., Liang L., Wu Z., Zhang H., Huang L., Cao H. (2021). IGZO/CsPbBr_3_-nanoparticles/IGZO neuromorphic phototransistors and their optoelectronic coupling applications. ACS Appl. Mater. Interfaces.

[121] Kim J., Kim Y., Kwon O., Kim T., Oh S., Jin S., Park W., Kwon J., Hong S., Lee C. (2020). Modulation of synaptic plasticity mimicked in al nanoparticle-embedded IGZO synaptic transistor. Adv. Electron. Mater..

[122] Kim H.-S., Park H., Cho W.-J. (2023). Light-Stimulated IGZO Transistors with Tunable Synaptic Plasticity Based on Casein Electrolyte Electric Double Layer for Neuromorphic Systems. Biomimetics.

[123] Sun J., Oh S., Choi Y., Seo S., Oh M.J., Lee M., Lee W.B., Yoo P.J., Cho J.H., Park J. (2018). Optoelectronic synapse based on igzo-alkylated graphene oxide hybrid structure. Adv. Funct. Mater..

[124] Jang J., Park S., Kim D., Kim S. (2024). Synaptic plasticity and associative learning in IGZO-based synaptic transistor. Sens. Actuators A Phys..

[125] Park J., Jang Y., Lee J., An S., Mok J., Lee S. (2023). Synaptic Transistor Based on In-Ga-Zn-O Channel and Trap Layers with Highly Linear Conductance Modulation for Neuromorphic Computing. Adv. Electron. Mater..

[126] Park H., Oh S., Jeong S.-H., Kwon O., Seo H.Y., Kwon J.-D., Kim Y., Park W., Cho B. (2022). Dual-Terminal Stimulated Heterosynaptic Plasticity of IGZO Memtransistor with Al2O3/TiO2 Double-Oxide Structure. ACS Appl. Electron. Mater..

[127] Ouyang W., Teng F., He J., Fang X. (2019). Enhancing the photoelectric performance of photodetectors based on metal oxide semiconductors by charge-carrier engineering. Adv. Funct. Mater..

[128] Abbas S., Kim J. (2020). All-metal oxide transparent photodetector for broad responses. Sens. Actuators A Phys..

[129] Alsaif M.M.Y.A., Kuriakose S., Walia S., Syed N., Jannat A., Zhang B.Y., Haque F., Mohiuddin M., Alkathiri T., Pillai N. (2019). 2D SnO/In_2_O_3_ van der Waals heterostructure photodetector based on printed oxide skin of liquid metals. Adv. Mater. Interfaces.

[130] Dodda A., Oberoi A., Sebastian A., Choudhury T.H., Redwing J.M., Das S. (2020). Stochastic resonance in MoS_2_ photodetector. Nat. Commun..

[131] Liao F., Deng J., Chen X., Wang Y., Zhang X., Liu J., Zhu H., Chen L., Sun Q., Hu W. (2020). A Dual-Gate MoS_2_ Photodetector Based on Interface Coupling Effect. Small.

[132] Yoshioka K., Wakamura T., Hashisaka M., Watanabe K., Taniguchi T., Kumada N. (2022). Ultrafast intrinsic optical-to-electrical conversion dynamics in a graphene photodetector. Nat. Photonics.

[133] Bai F., Qi J., Li F., Fang Y., Han W., Wu H., Zhang Y. (2018). A high-performance self-powered photodetector based on monolayer MoS_2_/Perovskite heterostructures. Adv. Mater. Interfaces.

[134] Jing H., Peng R., Ma R.-M., He J., Zhou Y., Yang Z., Li C.-Y., Liu Y., Guo X., Zhu Y. (2020). Flexible ultrathin single-crystalline perovskite photodetector. Nano Lett..

[135] Leung S., Ho K., Kung P., Hsiao V.K.S., Alshareef H.N., Wang Z.L., He J. (2018). A self-powered and flexible organometallic halide perovskite photodetector with very high detectivity. Adv. Mater..

[136] Huang J., Lee J., Vollbrecht J., Brus V.V., Dixon A.L., Cao D.X., Zhu Z., Du Z., Wang H., Cho K. (2020). A high-performance solution-processed organic photodetector for near-infrared sensing. Adv. Mater..

[137] Wu Y., Fukuda K., Yokota T., Someya T. (2019). A highly responsive organic image sensor based on a two-terminal organic photodetector with photomultiplication. Adv. Mater..

[138] Chow P.C.Y., Someya T. (2020). Organic photodetectors for next-generation wearable electronics. Adv. Mater..

[139] Lee I.S., Jung J., Choi D.H., Jung S., Kwak K., Kim H.J. (2021). Novel method for fabricating visible-light phototransistors based on a homojunction-porous IGZO thin film using mechano-chemical treatment. ACS Appl. Mater. Interfaces.

[140] Rho H.Y., Bala A., Sen A., Jeong U., Shim J., Oh J.O., Ju Y., Naqi M., Kim S. (2023). Plasma-engineered amorphous metal oxide nanostructure-based low-power highly responsive phototransistor array for next-generation optoelectronics. ACS Appl. Nano Mater..

[141] Jeon Y., Seo J., Yoo H. (2023). Air-stable ambipolar charge transport behaviors of organic-inorganic hybrid bilayer and application to Au nanoparticle-based floating gate memory. J. Alloys Compd..

[142] Lenef J.D., Jo J., Trejo O., Mandia D.J., Peterson R.L., Dasgupta N.P. (2021). Plasma-enhanced atomic layer deposition of p-type copper oxide semiconductors with tunable phase, oxidation state, and morphology. J. Phys. Chem. C.

[143] Han Y., Lee S., Kim M., Shin W., Lee H.K., Koo R., Lee S., Kim C., Yoo H. (2024). Charge Transport Advancement in Anti-Ambipolar Transistors: Spatially Separating Layer Sandwiched between N-Type Metal Oxides and P-Type Small Molecules. Adv. Funct. Mater..

[144] Jang Y., Lee S.-Y. (2022). In Situ IGZO/ZnON Phototransistor Free of Persistent Photoconductivity with Enlarged Spectral Responses. ACS Appl. Electron. Mater..

[145] Yoo H., Kim W.-G., Kang B.H., Kim H.T., Park J.W., Choi D.H., Kim T.S., Lim J.H., Kim H.J. (2020). High photosensitive indium–gallium–zinc oxide thin-film phototransistor with a selenium capping layer for visible-light detection. ACS Appl. Mater. Interfaces.

[146] Yoo S., Kim D.S., Hong W.-K., Yoo J.I., Huang F., Ko H.C., Park J.H., Yoon J. (2021). Enhanced ultraviolet photoresponse characteristics of indium gallium zinc oxide photo-thin-film transistors enabled by surface functionalization of biomaterials for real-time ultraviolet monitoring. ACS Appl. Mater. Interfaces.

[147] Zhang C., Yin X., Chen G., Sang Z., Yang Y., Que W. (2023). High-performance photodetector with a-IGZO/PbS quantum dots heterojunction. ACS Photonics.

[148] Yu J., Javaid K., Liang L., Wu W., Liang Y., Song A., Zhang H., Shi W., Chang T.-C., Cao H. (2018). High-performance visible-blind ultraviolet photodetector based on IGZO TFT coupled with p–n heterojunction. ACS Appl. Mater. Interfaces.

[149] Wei S., Wang F., Zou X., Wang L., Liu C., Liu X., Hu W., Fan Z., Ho J.C., Liao L. (2020). Flexible quasi-2D perovskite/IGZO phototransistors for ultrasensitive and broadband photodetection. Adv. Mater..

[150] Sen A., Park H., Pujar P., Bala A., Cho H., Liu N., Gandla S., Kim S. (2022). Probing the efficacy of large-scale nonporous IGZO for visible-to-NIR detection capability: An approach toward high-performance image sensor circuitry. ACS Nano.

[151] Chen T., Wang C., Yang G., Lou Q., Lin Q., Zhang S., Zhou H. (2023). Monolithic Integration of Perovskite Photoabsorbers with IGZO Thin-Film Transistor Backplane for Phototransistor-Based Image Sensor. Adv. Mater. Technol..

[152] Wang Y., Wang L., Liu F., Peng Z., Zhang Y., Jiang C. (2020). Organic-inorganic hybrid heterostructures towards long-wavelength photodetectors based on InGaZnO-Polymer. Org. Electron..

